# Multivariable modeling: A retrospective cohort study exploring the impact of socioeconomic status and distance to a rural academic center on all-cause preterm delivery

**DOI:** 10.1371/journal.pone.0306859

**Published:** 2024-10-03

**Authors:** Shilpa Darivemula, Marilyn Kouassi-Brou, Catherine Pollack, Amy Paris, Daisy Goodman, Timothy Fisher

**Affiliations:** 1 Department of Obstetrics and Gynecology, Dartmouth-Hitchcock Medical Center, Lebanon, New Hampshire, United States of America; 2 Department of Undergraduate Medical Education, Geisel School of Medicine, Hanover, New Hampshire, United States of America; 3 Department of Epidemiology, Graduate School of Quantitative Biomedical Sciences, Dartmouth College, Hanover, New Hampshire, United States of America; The Aga Khan University Hospital (AKUH), PAKISTAN

## Abstract

**Objective:**

Hospital-based labor and delivery units are closing at increasing rates in the rural US, significantly impacting maternal and newborn health. The objective of this study to determine if rurality—measured as distance from the hospital—and socioeconomic status—measured as insurance payor—impact both spontaneous and medically indicated preterm birth incidence at a single rural academic institution.

**Methods:**

This was a retrospective cohort study using electronic medical records of patients with singleton pregnancies delivering at a single rural academic institution between 2016–2018. The primary outcome was preterm delivery (PTD) and secondary outcomes included low birth weight (LBW) and intensive care nursery (ICN) admission. The primary exposures included (1) travel time from a patient’s address to the hospital and (2) insurance carrier as a proxy for socioeconomic status. Bivariate analyses indicated that travel time, insurance status, race, ethnicity, marital status, number of prenatal visits, gravida and para, and smoking status were significant predictors of all outcomes (LBW, ICN admission, and PTD). Therefore, these predictors were included in the multivariable logistic models.

**Results:**

Within the multivariable logistic model, patients traveling 1–1.5 hours had approximately twice the odds of PTD (Odds Ratio, OR: 2.08, 95% Confidence Interval CI, 1.32, 3.29, p = .002), birth of a LBW neonate (OR: 2.15; 95% CI: 1.29–3.58, *p* = .005), and infant admission to the ICN (OR 1.83, 95% CI: 1.22–2.76, *p* = .004) compared to patients traveling under 30 minutes,. Insurance carrier status was not associated with increased odds of PTD, LBW, or ICN admission.

**Conclusion:**

Patients living 1-to-1.5 hours from the hospital had an increased risk for LBW, ICN admission, and PTD, despite living in zip codes with less social deprivation than zip codes further away from the hospital.

## Introduction

Preterm delivery (PTD), defined as delivery prior to thirty-seven weeks gestational age, is a marker of obstetrical inequity and the leading cause of infant mortality [1–4]. Between 2011 and 2015, about eight percent of rural labor and delivery units closed their doors due to poor reimbursement policies, high costs of childbirth, low patient volume, and the lack of rural providers [5]. As of 2014, more than half of rural counties in the Unites States were described as maternity care deserts, with limited prenatal care and no hospital-based labor and delivery services [5]. Rural counties with a larger population of non-Hispanic Black women of reproductive age had higher odds of closure, exacerbating existing disparities in maternal and infant outcomes for rural Black women when compared to their rural White counterparts [5]. In the first year after the loss of hospital–based obstetric services in rural counties, there was an increased likelihood of having both nonlocal and preterm births, increased maternal mortality, and increased rural infant mortality rates [6–8]. In New Hampshire, nine out of sixteen rural hospitals have closed since the year 2000, with two units closing in early 2018 [9]. Such closures have increased travel times, worsened all-cause birth outcomes, and decreased frequency of prenatal visits [9], especially for patients using Medicaid (S1 Fig). Medicaid covers nearly half of all US rural births; poor reimbursement packages to rural hospitals have furthered the progression of unit closures [5].

Interestingly, a deluge of rural hospital closures in the 1980s led to the development of critical access hospital designations by Centers for Medicaid and Medicaid Services (CMS); this designation was made to reduce the financial vulnerability and improve rural hospital access. However, there is no requirement for Critical Access Hospitals to include labor and delivery units. This disparity in policy allows for continued closure of these maternity care units. Recent recommendations from the Alliance on Innovation on Maternal Health (AIM) include conducting internal institutional data review to assess obstetrical outcomes and identify gaps in rural maternal health equity. While there are several studies assessing the impact of closures on preterm birth incidence, there are no current studies assessing the impact of these successive closures on birth outcomes at a single rural academic tertiary care center. The objective of this study was to examine associations between clinical, sociodemographic, and geographic risks factors with preterm birth, low birth weight, and neonatal intensive care unit admissions at a single rural level III Maternal Obstetric Care unit serving northern New England using multivariable modelling. We hypothesize that insurance carrier status—a proxy for socioeconomic status—and increasing distance from the hospital will be associated with an increased odds of all three outcomes in a rural population. Given the changing obstetrical landscape of rural New Hampshire and current AIM recommendations, analyzing preterm birth delivery offers an essential analysis to identify patients at risk for poor birth outcomes, to identify local barriers to access, and to inform institution-wide initiatives to address rural maternal equity at our institution.

## Materials and methods

This study was a retrospective electronic medical record cohort study of singleton pregnancies delivering at a single rural academic institution in the US between 2016 and 2018 for analysis between 2020 and 2021. This two-year period coincides with the implementation of ICD-10 diagnostic coding and occurs after the closure of seven regional obstetrical units in New Hampshire [9–11]. No OB units were closed during the study period, offering a period of stability to assess associations. Inclusion criteria includedthose with spontaneously conceived, singleton pregnancies at or greater than 20 weeks gestation delivering between 1/1/2016 to 12/31/2018. Exclusion criteria included multiple gestation, delivery prior to 20 weeks gestational age, and use of in-vitro fertilization for pregnancy (given that it is a confounder for preterm birth). Records were identified for review through a list generated by the Analytics Institute based on claims data (ICD-10 and current procedural terminology (CPT) codes) at a single institution. Chart review was performed by two reviewers to collect all pertinent information and verify billing data. Demographic, clinical (ICD-10 codes), and geographic data were abstracted from these charts (S1 Table).

After extensive literature review, these covariates were determined as impactful on preterm delivery.

### Exposures

The exposures of interest were travel time and insurance carrier. Travel time was calculated as the time taken to travel on a road network between a patient’s home address and DHMC using Google maps, which was then aggregated into 30-minute intervals (i.e., under 30 minutes, 30 minutes-to-1-hour, 1-to-1.5 hours, and over 1.5 hours) based on access guidelines from the American College of Obstetricians and Gynecologists [12]. Insurance was measured as a categorical variable of either commercial, Medicaid, Medicare, other, or government. Address datasets were anonymized and analyzed separately from the remaining protected health information to protect patient privacy.

### Outcomes

The primary outcome of interest was PTD, which was coded as a binary variable of either 37 or more weeks gestational age (not preterm) or under 37 weeks gestational age (preterm). The secondary outcomes of interest were low birth weight (LBW) and intensive care nursery (ICN) admission. LBW was a binary variable defined as equal to or below 2500 grams. ICN admission was also considered as a binary variable.

### Clinical and sociodemographic covariates

Clinical and sociodemographic covariates considered for each model are defined in S1 Table. Variables were selected based on literature review and clinical expertise, with emphasis placed on studied chronic diseases in rural settings. Variables such as type 1 diabetes, renal disease, and autoimmune diseases were not included given their clinically known increased risk for medically indicated preterm birth. Race was initially considered as a multi-category variable but later collapsed to White vs. Other given the small proportion of individuals of color individuals in the data (n = 171, 5.66%). Prenatal visit count was transformed to a binary variable of under 6 visits versus at least 6 visits, per World Health Organization guidelines (14). Gravida & para was also collapsed to a binary variable of either G1P0 or not G1P0. Spontaneous abortions were collapsed to a categorical variable of under 2 or at least 2. Body mass index (BMI) was categorized into normal weight (BMI below 25 kg/m^2^), overweight (BMI at least 25 and less than 30 kg/m^2^), and obese (BMI at least 30 kg/m^2^). Transfer status was also included a binary variable for whether the patient was transferred to DHMC. Inclusion of transfer status is based on clinical experience. Transfer status may confound the relationship between the exposure and outcome, since transfer patients have more complex clinical needs requiring access to high-risk obstetricians and Level III NICU and thus may be further from the primary hospital.

### Geographic covariates

Two administrative, zip code-level data sets were combined with zip code level data collected from the medical records to evaluate external geospatial factors that may influence the outcomes of interest. One geospatial factor was rurality, which was determined through the rural-urban commuting area (RUCA) codes from the US Department of Agriculture [13]. The 33 RUCA codes were collapsed into four categories (urban, large rural, small rural, and isolated) in accordance with pre-existing guidelines [14].

The other geographic measure of interest was social deprivation index (SDI), which was derived by the Robert Graham Center. SDI is derived from 7 measures collected by the US Census Bureau American Community Survey: income, education, employment, housing, household characteristics, and transportation [15]). These measures are used to generate a single value from 1 to 100, whereby 1 is the most deprived and 100 is the least deprived.

Potential sources of bias include the use of a single institution for chart abstraction and the use of the straight-line distance using Google Maps, which can miss road conditions and realities of travelling to the hospital. Addressing this bias involved inclusion of RUCA codes to address the landscape factors impacting driving distance. Given the exploratory retrospective nature of the study, a sample size for power was not calculated. Any missing data variables in data abstraction led to the removal of the incomplete data.

### Statistical analysis

Bivariate analysis was conducted for each of the candidate covariates to determine which were independently and significantly associated with each of the outcomes of interest (i.e., PTD, LBW, and ICN). Variables that were significant in the bivariate analysis were included in corresponding multivariable analysis. Regardless of bivariate significance, SDI and rurality were included in each multivariable following a review of the literature. All models were mixed-effects logistic regression models with a random intercept for zip code to account for possible clustering effects. Variance inflation factor (VIF) was calculated for parameters in each multivariable model to assess the potential for multi-collinearity; all variables had a VIF below 5, suggesting that severe multi-collinearity was not present within the models. For each model, a *P*-value of less than 0.05 was considered significant. Analysis was conducted in R (version 4.1.2) in the RStudio Integrated Development Environment (version 2021.09.0).

### Ethics statement

The study was reviewed by the institutional review board at Dartmouth-Hitchcock Medical Center, who waived the requirement for informed consent. To protect patient privacy, all data were fully anonymized before team access and review.

## Results

### Study population

There were 3571 singleton deliveries between 2016 and 2018 at our single academic rural medical institution.

There were 126 deliveries excluded from analysis after failing to meet inclusion criteria due to missing data, multiple gestation, or use of artificial reproduction technologies (S2 Fig).

Demographic data is summarized in S2 Table.

The mean age of our study population was 29.6 (SD: 5.58), and a majority were White patients (94.3%), followed by Asian (3.87%), African American or Black (1.29%), and American Indian/Alaskan Native (0.5%). Of all patients, 64.7% (n = 1956) had commercial insurance, followed by Medicaid (32.6%), Medicare (1.62%), and other government insurance (1.03%). While most patients were obese (56.7%), the prevalence of other chronic diseases was low; only 5.26% had chronic hypertension, 10.5% had gestational diabetes, 2.55% had type 2 diabetes, and 10% had opioid use disorder. Most patients were multiparous and did not have a history of preterm delivery (68.2% and 97.5%, respectively). About half of the patients traveled between 30 minutes and 1 hour to receive care (42.7%), while a third were within 30 minutes of the hospital (29.8%) (S2 Table). Exactly 16.3% (n = 563) of deliveries were preterm, 14.3% (n = 494) were LBW neonates, and 17.6% (n = 608) required ICN admission.

### Geographic measures

Thirty percent of patients lived under 30 minutes from the hospital, compared to 16% who lived between 1 and 1–1.5 hours away. Communities located over 2 hours away from the hospital had a significantly higher SDI than those 1.5 hours away (*P* = .009) (S3 Fig).

There was no significant difference in SDI between those living between 1 to 1.5 hours from the hospital and those living within 30 minutes of the hospital ($\bar{x}$ = 5.06, 95% CI: -8.63, 18.8, *P* = .850). Patients on Medicare or Medicaid lived in zip codes with a higher SDI compared to those on commercial insurance (*P* < .001) (S4 Fig)

Most patients lived in a large rural area (34.9%), followed by isolated areas (31.5%), small rural (25.1%), and urban (8.47%). SDI was significantly different by rurality (*P* < .001). In particular, individuals who lived in small rural zip codes had a significantly higher SDI (i.e., were less deprived) than those living in urban, ($\bar{x}$ = 9.93, 95% CI: 0.25, 19.6, *P* = .041), large rural ($\bar{x}$ = 15.3, 95% CI: 4.57, 26.0, *P* = .001), and isolated ($\bar{x}$ = 15.9, 95% CI: 5.96, 25.9 *P* < .001) zip codes. Only 8% of the study population included transfers.

### Multivariable analysis

Bivariate analyses indicated that travel time, insurance status, race, ethnicity, marital status, number of prenatal visits, gravida and para, and smoking status were significant predictors of all outcomes (LBW, ICN admission, and PTD) included in the multivariable models (S3 Table).

Covariates included in the final model include marital status, number of prenatal visits, history of preterm labor, type 2 diabetes, gravida-para, smoking status, body-mass index, rurality, SDI, and transfer status. Patients traveling 1–1.5 hours had approximately twice the odds of PTD (Adjusted Odds Ratio, OR: 2.08, 95% CI, 1.32, 3.29, *P* = .002), birth of a LBW neonate (OR: 2.15; 95% CI: 1.29–3.58, *P* = .005), and infant admission to the ICN (OR 1.83, 95% CI: 1.22–2.76, *P* = .004). Marital status, prenatal visit count, history of PTD, primiparity, and tobacco exposure also increased risks for PTD, LBW, and ICN admission. Insurance carrier status and social deprivation indices were not associated with increased odds of PTD, LBW, or ICN admission (S4 Table).

Sample size for this study was limited for all outcomes when distributed by distance from the hospital (S5 Table). Notably, cases in the 30 minutes to 1 hour category and the greater than 1 hour category were comparable (S5 Table).

When comparing the individual attenuation of the RUCA code and social deprivation index variables on travel time impacting preterm birth, low birth weight, and neonatal intensive care unit admission (ICN admission), only RUCA codes significantly increased the odds ratio of all three outcomes (S6 Table).

The degree of rurality had a 23% reduction in effect of time on preterm labor, a 6% reduction in effect on low birth weight, and a 16% reduction in effect on ICN admission (S6 Table). Social deprivation was not significant, and overall, the greater than 1 hour distance to the hospital has a 65% increased risk for all three outcomes in models adjusted for other covariates and indicators of rurality (S6 Table).

## Discussion

Among births at our academic medical center, after controlling for transfer status, patients living 1-to-1.5 hours from the hospital had an increased risk for low birth weight, neonatal intensive care unit admission, and preterm birth, compared to those traveling under 30 min (S4 Table). A history of preterm birth and limited prenatal visits were also associated with an increased likelihood of all three adverse outcomes, consistent with previously published data on increased rurality and associated increased preterm birth rates (S4 Table). Although the degree of social deprivation increased in this population with increased distance and increased degree of rurality (S3 and S4 Figs), the degree of social deprivation and insurance carrier status—proxies for socioeconomic deprivation—were not associated with preterm birth, low birth weight or ICN admission in our population. Interestingly, it is the degree of rurality—the RUCA code—that attenuates the relationship between distance to the hospital and preterm birth rates, with statistically significant increases in odds ratios notes for all three outcomes when RUCA codes alone were applied (S6 Table). Rurality is a strong predictor on the risk of all cause preterm birth at our institution, emphasizing the importance of further study into the diverse aspects of New Hampshire rurality impacting this outcome.

Several studies have assessed the relationship between distance and rural maternal health outcomes. Grzybowski et al noted that the adjusted odds ratio for perinatal mortality for newborns from rural communities living greater than four hours away from the hospital was three times greater than those living closer to the hospital [16]. Other studies assessed area level deprivation and its association to maternal morbidity; While the studies looking at effect of distance on rural maternal outcomes determined a significant impact on rates of preterm birth [4,6], neither study explicitly measured the distance from the hospital and its direct correlation to PTB. When examining the distribution of travel times by rurality, most of the area within thirty minutes of the hospital of interest within this study was described as “large rural” per RUCA codes. Yet, patients living an hour away lived in urban, isolated, small rural and large rural communities and were at increased risk for all outcomes. This suggests that distance from the hospital and its impact on preterm deliveries, low birth weight, and ICN admissions is not based solely on degree of rurality, but also on how rurality impacts maternal care, such as road access to the hospital, to local resources, and to community and clinical support. Several of the hospital closures were within one to two hours distance from our academic institution, raising concern that proximity to hospitals is a major risk factor for poor obstetrical outcomes—a concern that is increasing as more hospital units close their obstetrical units [9]). Zip code tabulations are used for geographical factors, but do not capture cohesive areas and often mask the variety of resources, needs, and cultures living in these areas [17] Census tracts have also been used to measure rurality, but it does not follow city or county boundaries and thus runs the risk of rural-urban misclassification [17]. Area level deprivation indices do not always include covariates relevant to rural maternal care [17]. Defining rurality remains an epidemiological challenge and deserves further research to determine the specific resources and characteristics impacting rural maternal care [17].

Strengths of this study include creating local data to compare national and state-wide trends and including multiple variables to assess socioeconomic vulnerability, such as SDI, insurance, or distance. Our data is consistent with findings in published research which lends a level of generalizability to state wide data [18]. Limitations include the potential for unmeasured confounding due to missing covariates in the models and low power for each of the outcomes when divided by distance (S5 Table). Retrospective data collection is limited by inaccurate diagnostic coding and limited numbers of patients from remote areas of the state, impacting the selection and power of the covariates used in the models. For example, inclusion of other substance use disorders beyond opioid use would have enhanced clinical relevancy. Determining which preterm births were spontaneous versus medically indicated—a clinically important distinction—was impossible due to the generalized coding schema used and the retrospective nature of this study. Future studies should consider repeating this methodology at several rural institutions over time to improve the power of covariates and to consider prospective trials in the identified areas of high all-cause preterm birth rates to determine local causes for spontaneous preterm birth specifically.

One in three birthing people now live in an obstetric desert, and more than 50% of rural counties have no hospital-based obstetrical services, forcing rural birthing people to travel more than thirty minutes for care [19]. Hostetter and Klein describe grants, such as the Rural Maternity and Obstetrics Management Strategies, to create regional networks of care for rural people, with networks focusing on the specific cultural, socioeconomic, and geographic needs of their populations [19]. Understanding these granular differences and needs requires institutional data analysis; the Alliance for Innovation on Maternal Health recommends this institutional review of obstetrical data but offers little guidance on recommended best practices to collect and analyze this information. Despite having better socioeconomic deprivation scores, patients traveling from one to one and a half hours away seemed to have increased risk for preterm birth, low birth weight, and neonatal care unit admissions; this indicates the importance of conducting local needs assessments, developing rural maternity health indices to capture identified factors missed in the SDI measurement, and repeating these studies over time to determine if local interventions are improving outcomes for rural maternal health equity and safety. Our study offers a methodologic framework for the collection and analysis of birth outcomes data for a single institution, with the hopes of encouraging specific solutions for the specific local needs of highlighted communities. By identifying the patient catchment areas with high degrees of preterm birth, low birth weight and NICU admission, we hope to be able to be able to provide information for future clinical interventions in the setting of the changing rural maternal health landscape and present a way for individual institutions to assess their own outcomes for future quality improvement.

## Supporting information

S1 Fig Map of labor and delivery unit closures across Northern New England, marking closures after the year 2000. Squares represent labor and delivery unit closures. Circles represent open labor and delivery units. Triangles represent open freestanding birth centers. Reprinted from OpenStreetMap under a CC BY license, with permission from David LaFlamme, original copyright 2023.
(TIFF)

S2 Fig Frequencies of preterm birth, low birth weight, and neonatal intensive care unit admissions for the patient cohort. This flowchart describes the composition of each of the three outcomes.
(TIFF)

S3 Fig SDI score against travel time boxplot. This chart assessed the SDI score against travel time and noted that zip codes over 2 hours away from the hospital had a significantly higher SDI than zip codes 1–1.5 hours away (*P* = .009). Zip codes that are over 2 hours away had significantly higher SDI (more disenfranchised) than those 1–1.5 hours away (P = .009); 30 minutes-1 hour away (P = 0.002); and under 30 minutes (p = 0.026).
(TIFF)

S4 Fig SDI score against insurance type boxplot. This noted that patients on Medicare or Medicaid lived in zip codes that are more disenfranchised than people living in zip codes with commercial insurance. Patients on Medicare or Medicaid lived in zip codes with a higher SDI (more disenfranchised) compared to those on commercial insurance (P < 0.001).
(TIFF)

S1 Table Demographic, clinical and geographic covariates. (TIFF)

S2 Table Demographic characteristics. (TIFF)

S3 Table Bivariate analysis with potential covariates. (TIFF)

S4 Table Multivariable models. (TIFF)

S5 Table Distribution of cases/non cases per outcome. Cases and non-cases numerically listed for all three outcomes divided by distance.
(TIFF)

S6 Table Travel time estimate variation. Adjusted odds ratio attenuations of rurality, described by RUCA codes, and social deprivation index on the relationship between travel time and the three outcomes of interest.
(TIFF)
