# Supplementary figures and images for: Multivariable modeling: A retrospective cohort study exploring the impact of socioeconomic status and distance to a rural academic center on all-cause preterm delivery
Source: PLoS One. 2024 Oct 3;19(10):e0306859. doi: 10.1371/journal.pone.0306859 (PMC11449359; doi:10.1371/journal.pone.0306859)

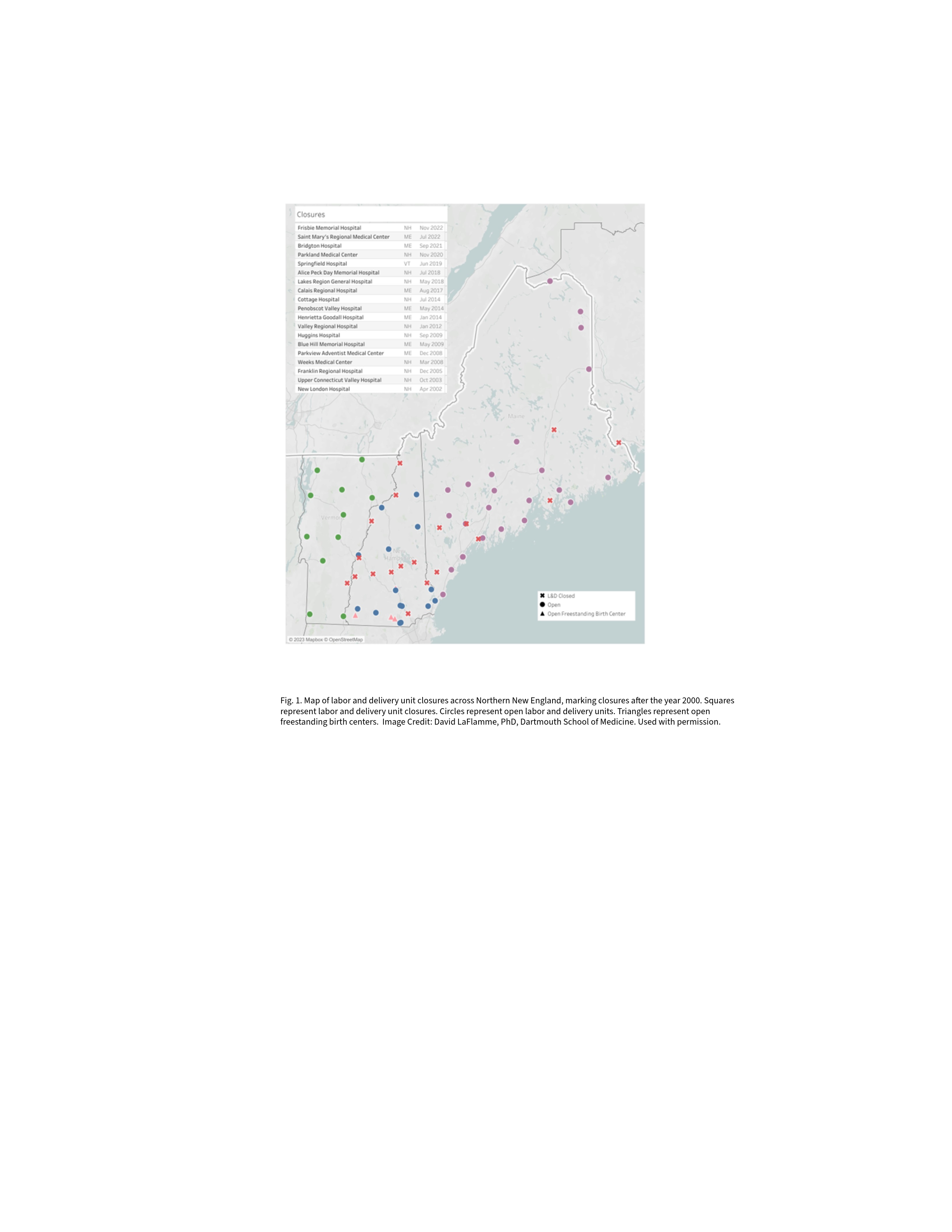

Supplement: S1 Fig — Squares represent labor and delivery unit closures. Circles represent open labor and delivery units. Triangles represent open freestanding birth centers. Reprinted from OpenStreetMap under a CC BY license, with permission from David LaFlamme, original copyright 2023. (TIFF) [file pone.0306859.s001.tiff]

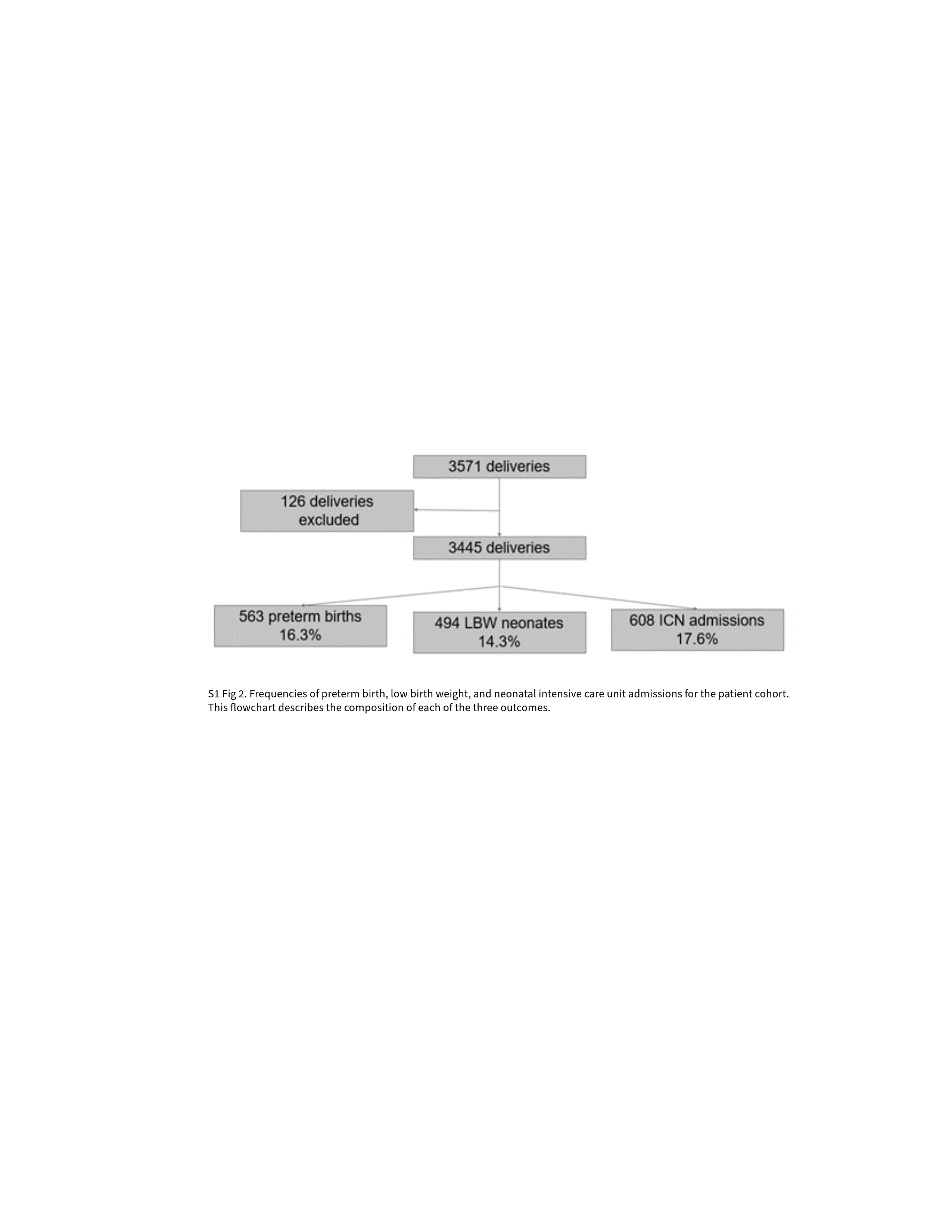

Supplement: S2 Fig — This flowchart describes the composition of each of the three outcomes. (TIFF) [file pone.0306859.s002.tiff]

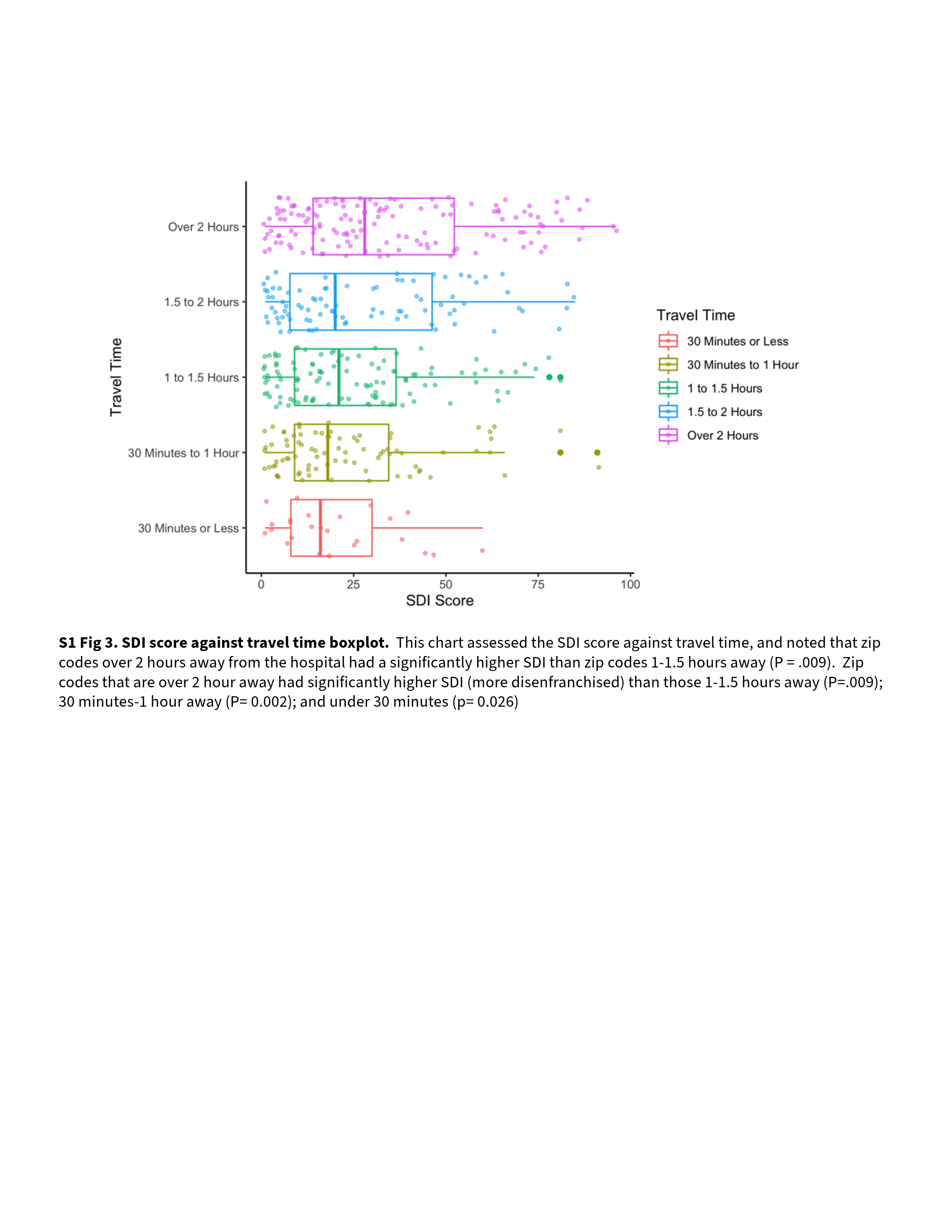

Supplement: S3 Fig — This chart assessed the SDI score against travel time and noted that zip codes over 2 hours away from the hospital had a significantly higher SDI than zip codes 1–1.5 hours away (P = .009). Zip codes that are over 2 hours away had significantly higher SDI (more disenfranchised) than those 1–1.5 hours away (P = .009); 30 minutes-1 hour away (P = 0.002); and under 30 minutes (p = 0.026). (TIFF) [file pone.0306859.s003.tiff]

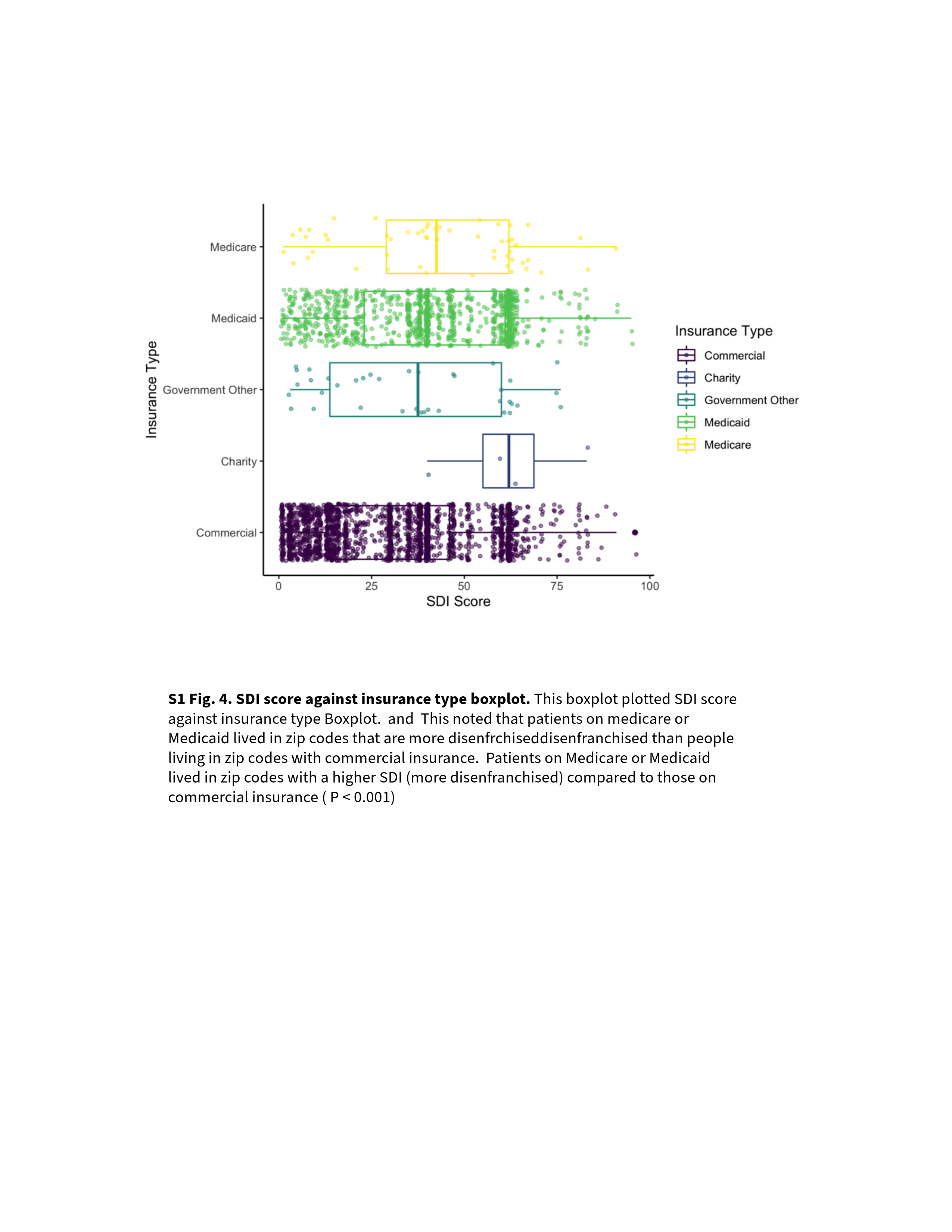

Supplement: S4 Fig — This noted that patients on Medicare or Medicaid lived in zip codes that are more disenfranchised than people living in zip codes with commercial insurance. Patients on Medicare or Medicaid lived in zip codes with a higher SDI (more disenfranchised) compared to those on commercial insurance (P < 0.001). (TIFF) [file pone.0306859.s004.tiff]

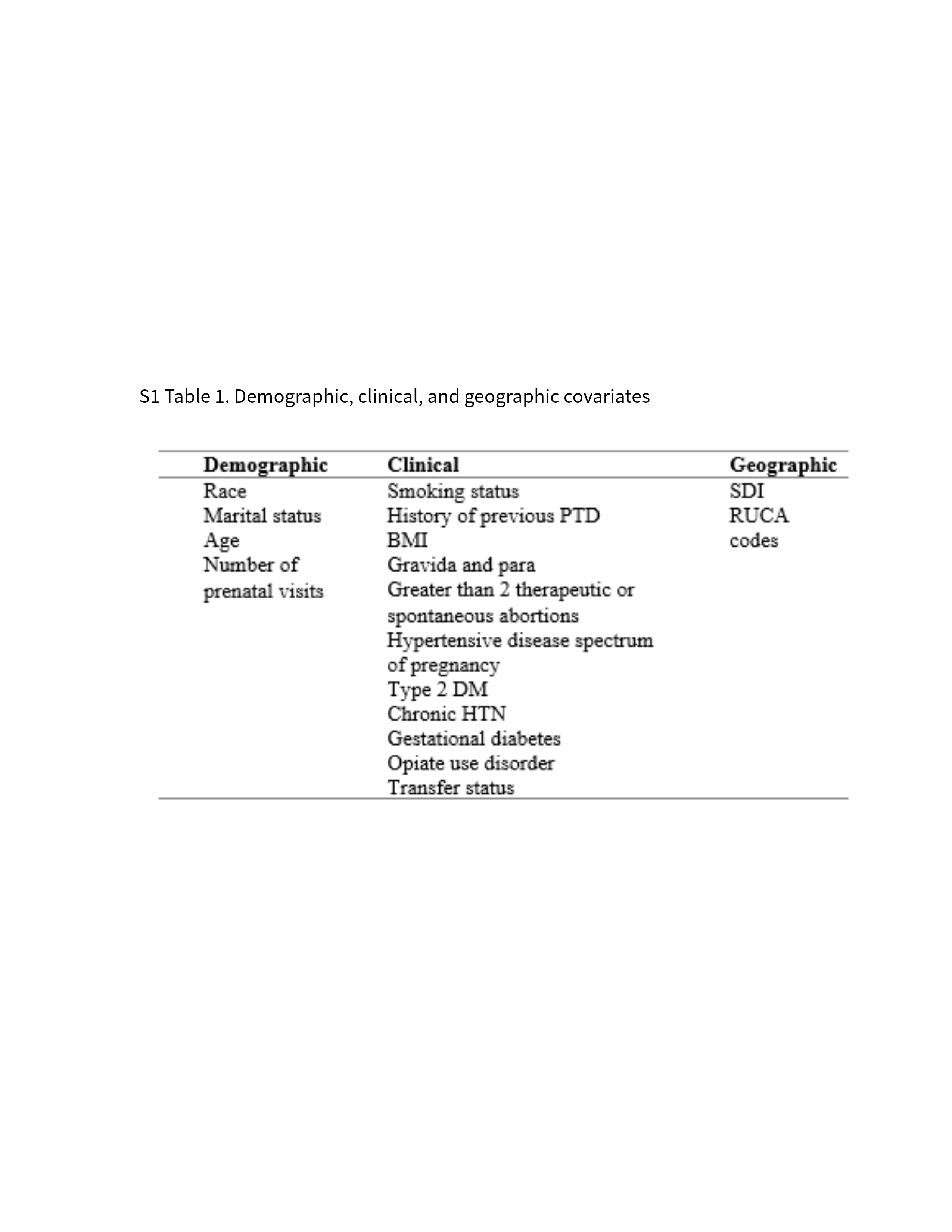

Supplement: S1 Table — (TIFF) [file pone.0306859.s005.tiff]

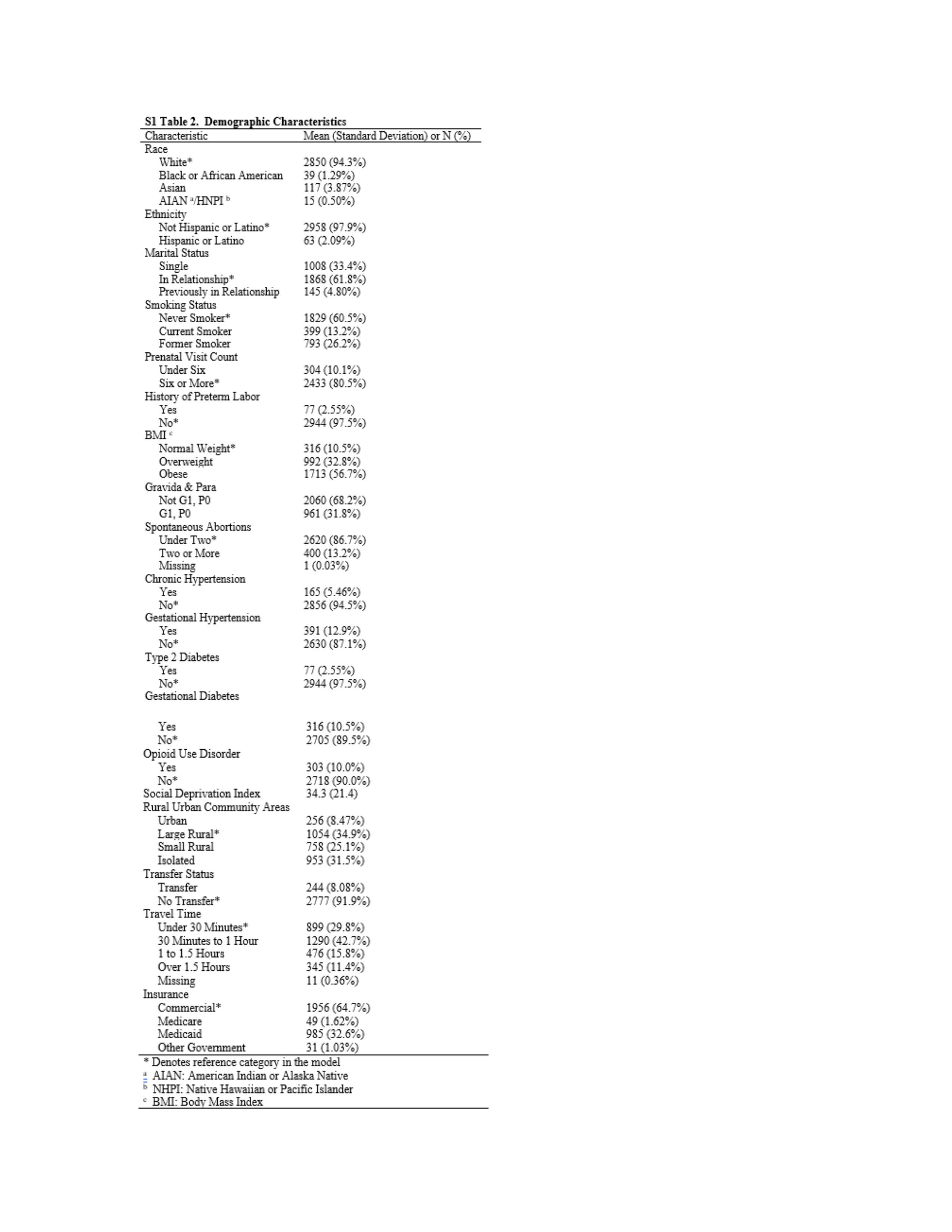

Supplement: S2 Table — (TIFF) [file pone.0306859.s006.tiff]

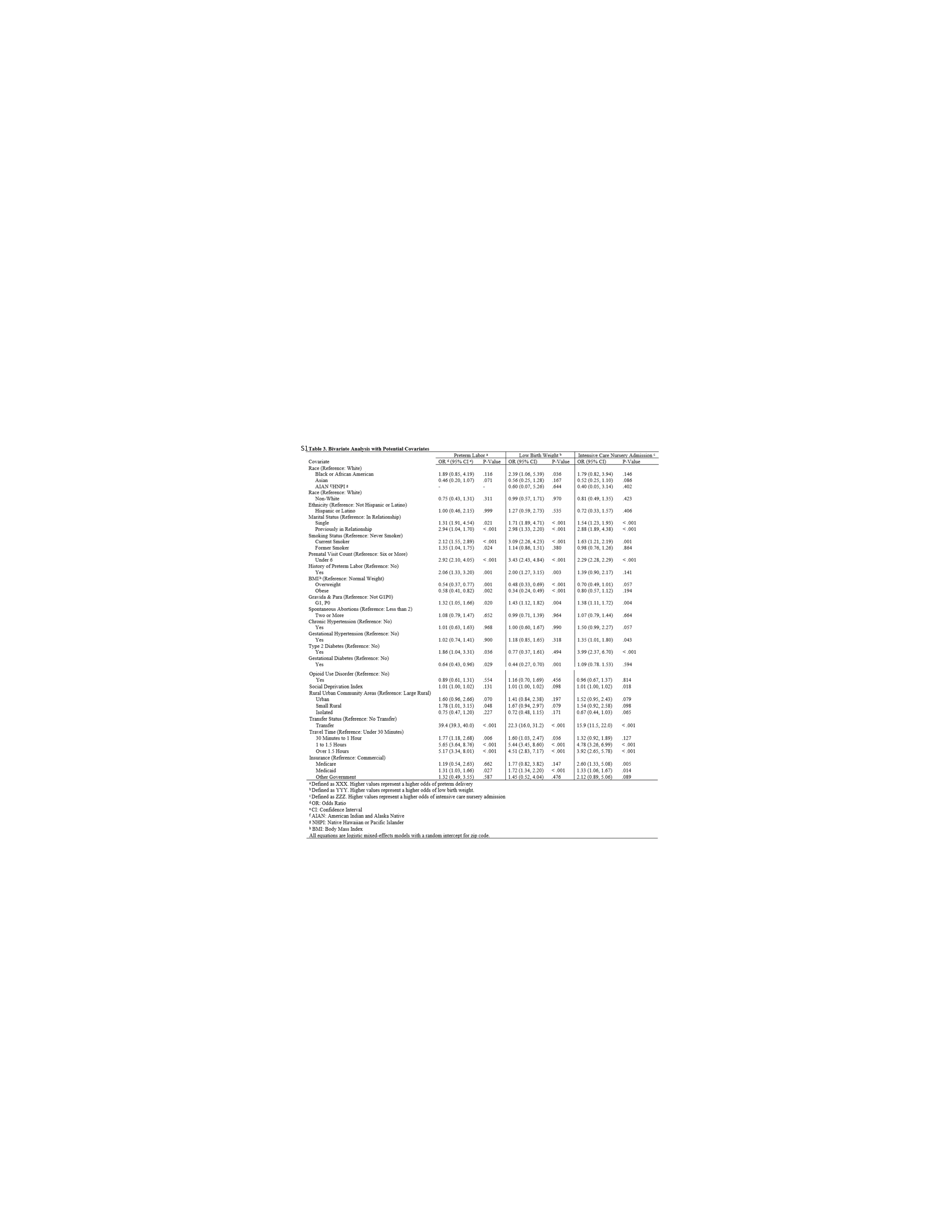

Supplement: S3 Table — (TIFF) [file pone.0306859.s007.tiff]

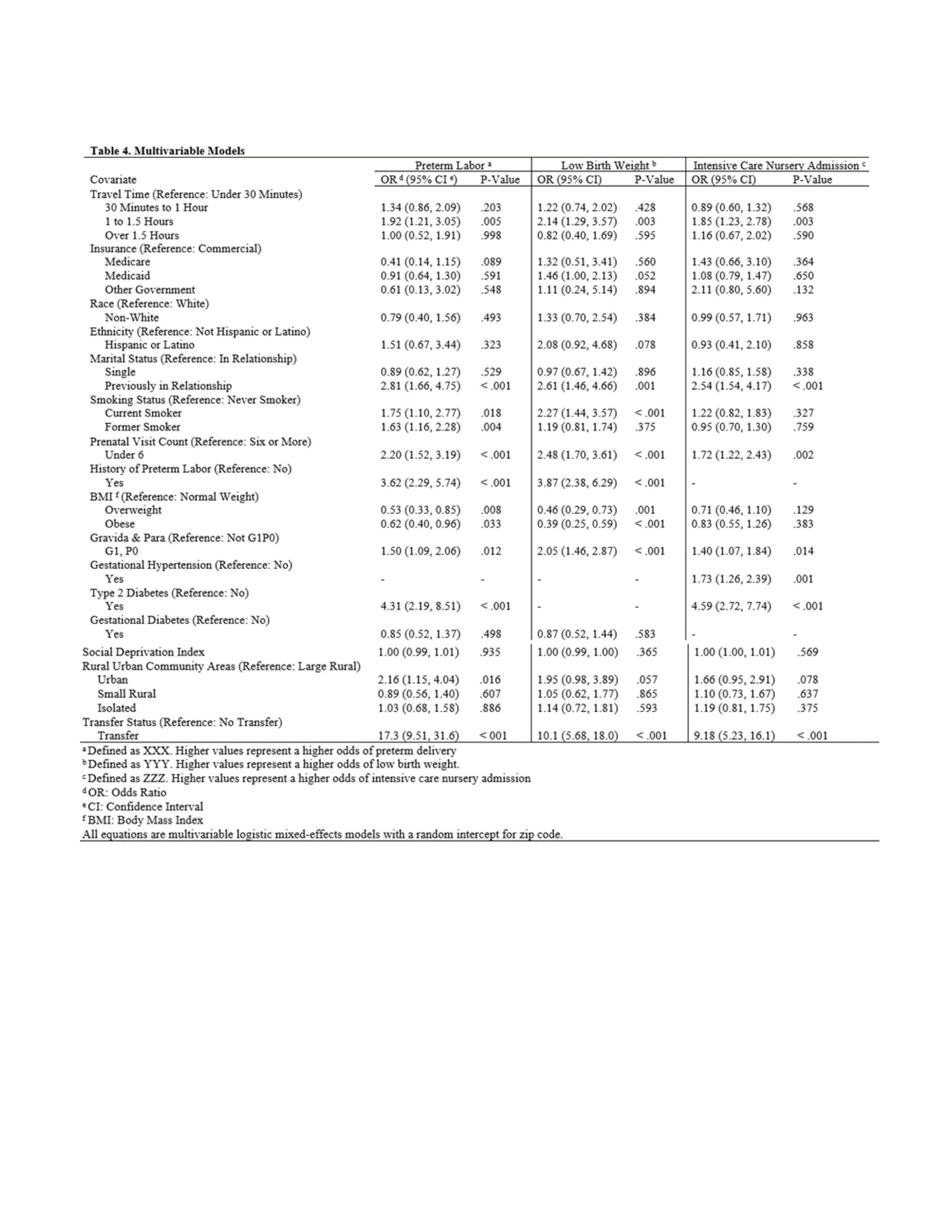

Supplement: S4 Table — (TIFF) [file pone.0306859.s008.tiff]

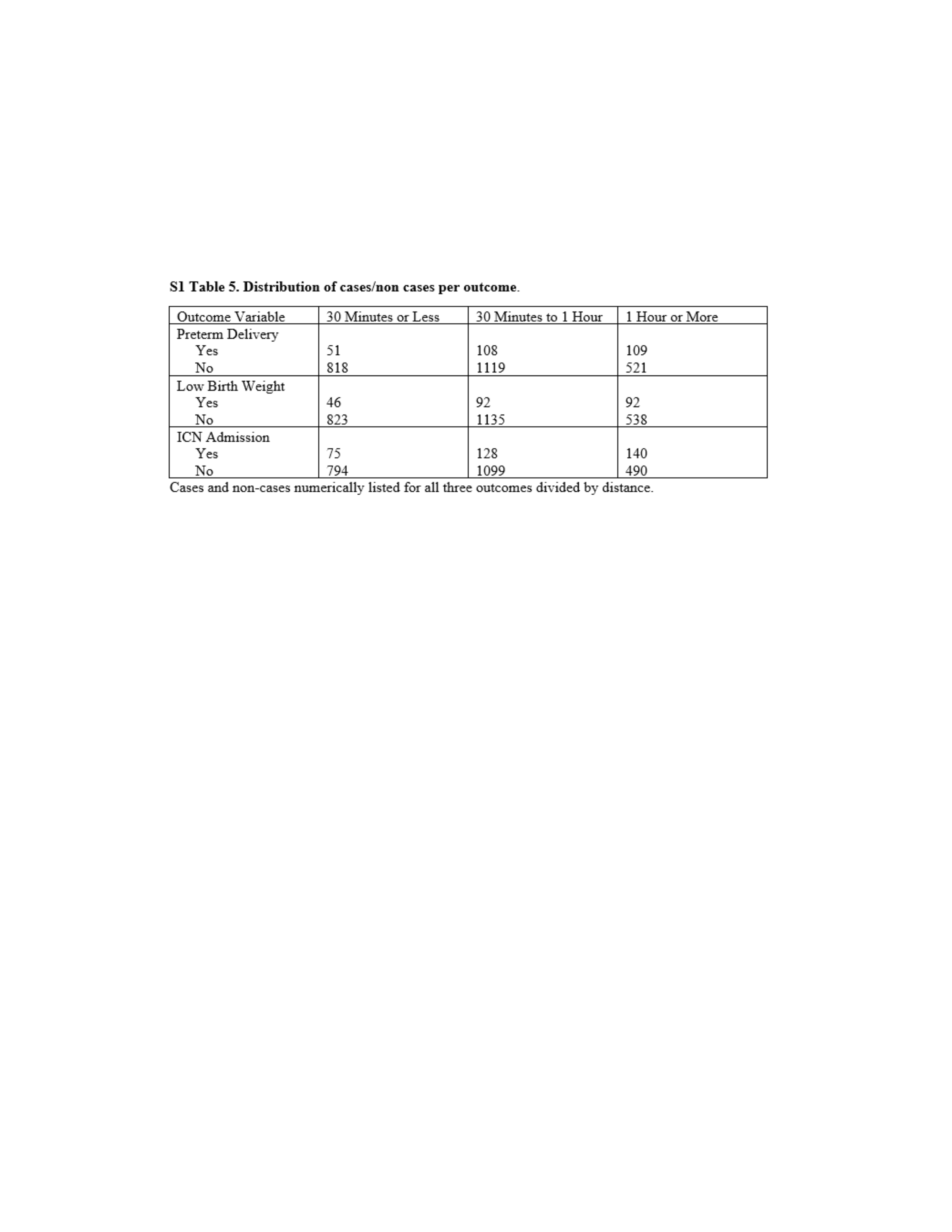

Supplement: S5 Table — Cases and non-cases numerically listed for all three outcomes divided by distance. (TIFF) [file pone.0306859.s009.tiff]

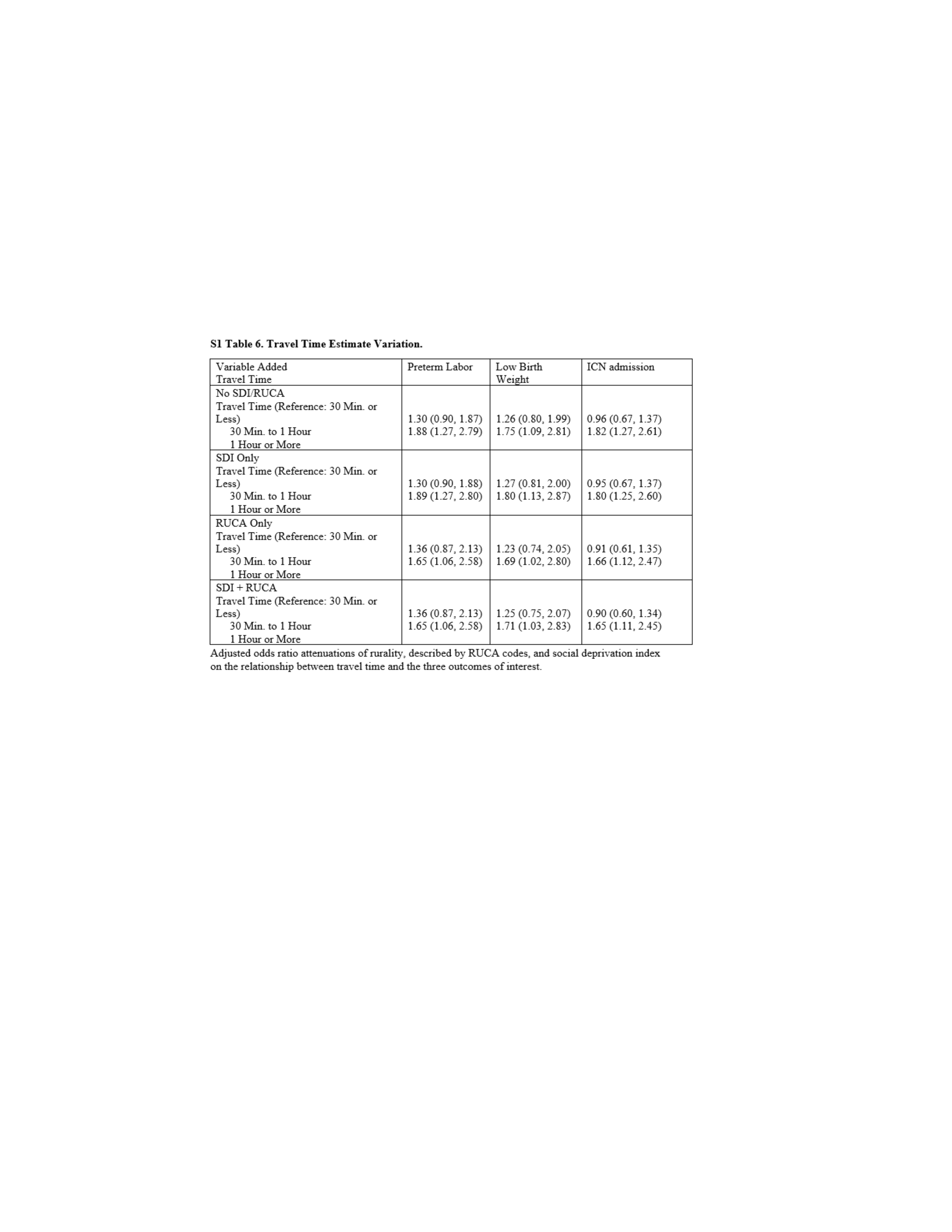

Supplement: S6 Table — Adjusted odds ratio attenuations of rurality, described by RUCA codes, and social deprivation index on the relationship between travel time and the three outcomes of interest. (TIFF) [file pone.0306859.s010.tiff]
